# Inflammatory role of dendritic cells in Amyotrophic Lateral Sclerosis revealed by an analysis of patients’ peripheral blood

**DOI:** 10.1038/s41598-017-08233-1

**Published:** 2017-08-10

**Authors:** Michela Rusconi, Francesca Gerardi, William Santus, Andrea Lizio, Valeria Ada Sansone, Christian Lunetta, Ivan Zanoni, Francesca Granucci

**Affiliations:** 1 Department of Biotechnology and Bioscience, University of Milano-Bicocca, Piazza della Scienza 2, 20126 Milan, Italy; 2 NEuroMuscular Omnicentre, Fondazione Serena Onlus, Milano, Italy; 3 Harvard Medical School and Division of Gastroenterology, Boston Children’s Hospital, Boston, MA 02115 USA; 4 Department Biomedical Sciences for Health, University of Milan, Milan, Italy

## Abstract

Chronic inflammation is one of the causes of neurodegeneration in Amyotrophic lateral sclerosis (ALS). Here we examined whether circulating dendritic cells (DCs) can contribute to disease progression. We found ALS patients show a significant reduction in the number of circulating DCs. Also, patients’ DCs present an increased expression of CD62L and a tendency to overexpress CCR2 compared with healthy donors. Moreover, DCs derived from a subpopulation of ALS patients produced higher levels of IL-8 and CCL-2 upon lipopolysaccharide (LPS)-stimulation. Finally, we found a significant inverse correlation between the time from onset of the pathology to its diagnosis and the levels of IL-6 secretion induced by LPS. Our data support the hypothesis, in a subpopulation of patients, DCs recruited at the diseased tissue produce high levels of CCL-2 and IL-8 and contribute to the inflammatory process promoting the recruitment of other inflammatory cells. An increased efficiency of IL-6 production may accelerate only the initial phases of disease progression. Blood DC analysis can be used to identify ALS patients with an altered course of inflammatory cell recruitment at the diseased central nervous system (CNS). The high levels of CD62L expression suggests this molecule could be a target for treatment of CNS inflammation.

## Introduction

Amyotrophic lateral sclerosis (ALS) is a lethal neurodegenerative disease, also known as Lou Gehrig’s disease, described for the first time by Jean Martin Charcot in 1800. It affects both upper (UMN) and lower motor neurons (LMNs) in the cortex, brainstem and spinal cord.

The name of the pathology resumes all the major features of the disease. “Amyotrophic” refers to muscular atrophy, and “lateral sclerosis” pertains to the scarring in the lateral aspect of the spinal cord^1^. ALS is a clinically heterogeneous disease characterized by muscles wasting, weakness, paralysis, swallowing impairment and respiratory failure that may occur in month or in years. These clinical features can also be accompanied or preceded by frontotemporal dementia^2^. Approximately 10% of ALS patients are familial and in the 20% of these cases the disease is caused by mutations in the gene encoding superoxide dismutase 1 (SOD1)^3, 4^. The cause of sporadic ALS (sALS) is incompletely defined and there is a consensus that pathogenesis is probably multifactorial.

Increasing evidence support the importance of chronic inflammation to drive neurodegeneration in ALS^5^. Spinal cord infiltration by peripheral proinflammatory monocytes, dendritic cells (DCs) and T cells has been found in patients and animal models^6^. Both CD4^+^ and CD8^+^ T lymphocytes infiltrates the brains of ALS patients and -by interacting with glial cells- could play a role in motor neuron degeneration. Also, DNA microarray analysis on postmortem spinal cord tissue obtained from individuals with sALS has revealed changes in the expression of genes involved in the inflammatory process^7, 8^. Similarly, autopsy of sALS patients and analyses of SOD1 transgenic mice have confirmed the idea that both innate and adaptive immunity are involved in the neurodegenerative process^9–11^.

Inflammation in ALS is not limited to the Central Nervous System (CNS) but is also present at the systemic level. This includes: increased numbers of circulating activated lymphocytes, higher levels of MHC class II expression on monocytes and higher levels of inflammatory serum cytokines. Patients with a pathology that rapidly progress also show low numbers of blood regulatory T cell (Tregs)^12^ and increased levels of lipopolysaccharide (LPS) in the plasma^13^. High endotoxin levels may contribute to chronic low-grade inflammation as manifested in chronic disease such as Parkinson’s disease and atherosclerosis^14^. Among the cells that respond to LPS, myeloid DCs are central players of the immune response. DCs decide of the activation of T cells through the presentation of the peptide–MHC complex (signal 1), the expression of costimulatory molecules (signal 2), and the production of cytokines (signal 3) activation^15^. The type of immune response induced by DCs strongly depends on the quality of the signals DC encounter^16^. In human peripheral blood, two main populations of DCs can be distinguished: CD11c^high^ myeloid DCs that are strongly activated by LPS, and CD11c^low^ plasmacytoid DCs. These DCs express distinct receptors for inflammatory stimuli and respond to different pathogenic stimuli, suggesting that each subset has a specialized function in directing T cells responses.

Because of their unique capacity to shape immune responses, DCs play important roles in the pathogenesis of several inflammatory diseases including neuroinflammatory disorders^17^. Studies on human DCs demonstrate that in the peripheral blood of patients with progressive neurodegeneration, these cells are functionally altered and more prone to skew T cell responses towards a proinflammatory phenotype^18^. In ALS, there is evidence that a transition from a neuroprotective Treg infiltrate to a deleterious Th1 response characterizes the increase in disease severity^19^.

Although DCs play a major role in shaping immune responses, their phenotype and functional features in peripheral blood of ALS patients have not been investigated. Understanding the possible alteration in the functionality of DCs in ALS patients compared to healthy subjects or patients affected by other neurodegenerative disorders could therefore represent an important step toward the definition of the inflammatory process that contributes to the progression of the disease. This could have important implications to define the prognosis and to select ALS patients potentially more responsive to therapeutic strategies for modifying the inflammatory state to restore the normal immune conditions.

Here we have investigated the functionality of peripheral blood myeloid DCs in ALS patients and correlated it with patients’ clinical state. A large cohort of ALS patients has been enrolled in the present study. *Ex vivo* analyses of the frequency and expression of costimulatory, MHC and migratory molecules of the major myeloid DC subset have been performed and the capacity of purified DCs to produce cytokines has been investigated *in vitro*.

This study provides a detailed picture of the functional state of blood DCs in ALS patients.

## Results

### Patients enrolled in the study

ALS patients enrolled in the study showed the presence of combined UMN and LMN involvement in at least one body region, and were classified with definite or probable diagnosis according to the revised El Escorial criteria^20^. We studied in total 72 patients, 20 for flow cytometry analyses and 52 for cytokine expression, all with sporadic ALS. Supplementary Tables 1–6 resume the characteristics of ALS patients and controls enrolled in the study.

The expression of the disease was assessed based on the ALS-Functional Rating Scale-Revised (ALS-FRS-R) and the definition was as follows: severe, with ALS-FRS-R score lower than 28; mild, with ALS-FRS-R score ranged from 29 to 43; and slight, with ALS-FRS-R score upper than 44. Rapidity of disease progression was assessed using the ratio between the score lost on the ALS-FRS-R and the disease duration: Progression index = (48 − ALS-FRS-R score)/duration in months.

Healthy Donors (n = 37, HD) and non-ALS Neurological Patients (n = 25, other neurological diseases, OND) stratified for age and sex were also enrolled as control.

### ALS patients show decreased numbers of circulating DCs that express higher levels of CD62L compared to healthy controls

**Figure 1:**
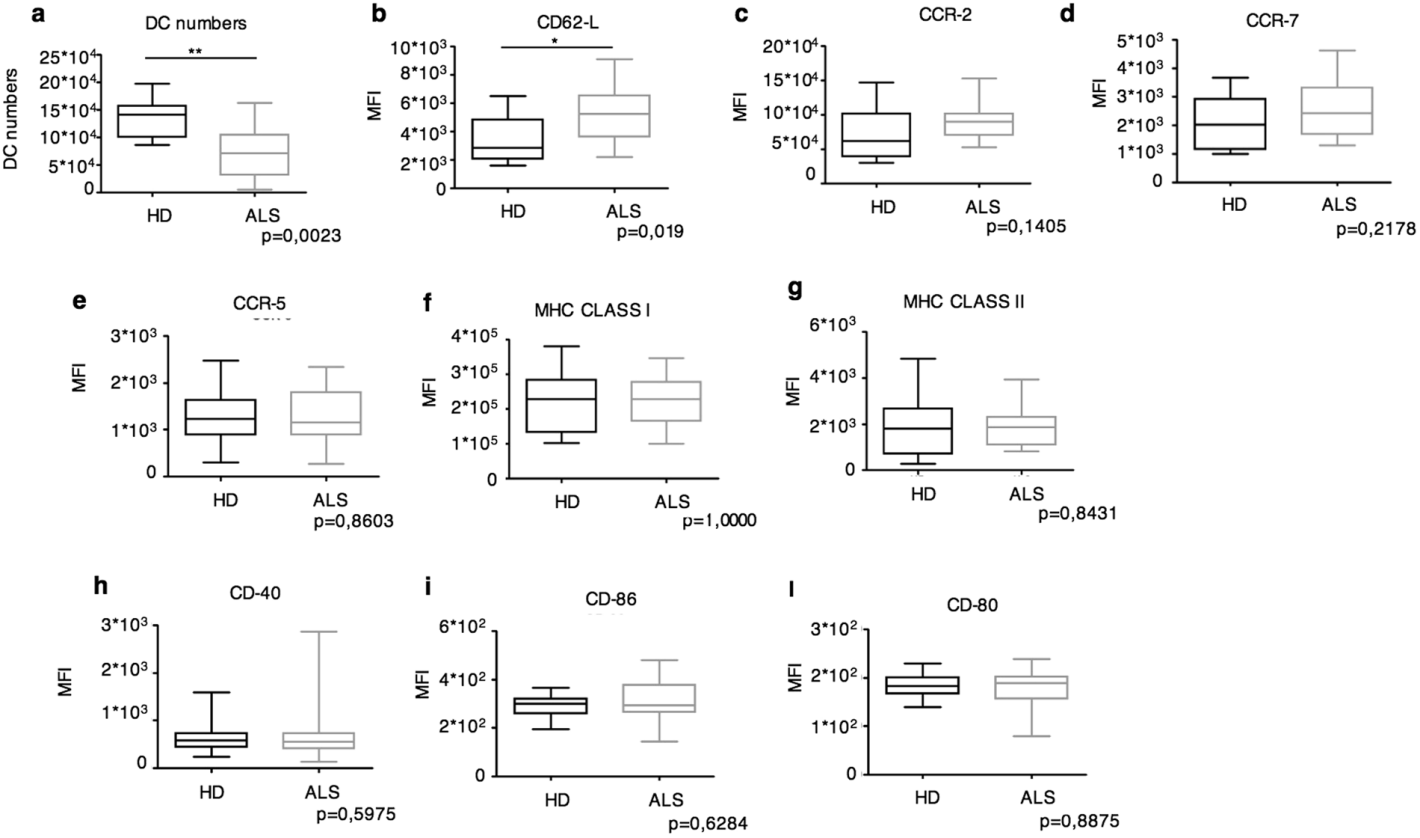
Phenotypic analysis of peripheral blood DCs in ALS patients compared to HD. (**a**) Box plots of absolute numbers of CD1c^+^CD19^−^ DCs in the peripheral blood of ALS patients (ALS, n = 20) and HD (n = 10). (**b**–**l**) Box plots of the Mean Fluoresce Intensity (MFI) of the indicated molecules in ALS patients compared to HD. *P < 0.05; **P < 0.01.

The frequency of myeloid DCs was analyzed in peripheral blood of ALS patients and healthy donors (HD). Characteristics of patients and HD used for this analysis are resumed in Supplementary Tables 1 and 2. In the blood, CD1c^high^ cells represent the major subset of myeloid DCs^21^. High amounts of CD1c are expressed also by a subset of small resting B cells, therefore we identified myeloid DCs as CD1c^high^CD19^−^. As shown in Fig. 1a, ALS patients had significantly less circulating DCs than HD.

Next, we examined the expression of activation markers (MHC and the costimulatory molecules CD80, CD86, CD40), and molecules involved in DC migration (CD62L, CCR5, CCR7 and CCR2). No differences were observed in the expression of MHC and costimulatory molecules, as well as the CCR5 and CCR7 chemochines (Fig. 1a,d–l). An increased trend of expression was, instead, observed for CCR2 and a significantly higher CD62L expression was measured on DCs from ALS patients compared to HD (Fig. 1b,c).

CD62L is a cell adhesion molecule required for the recruitment of the innate and adaptive cells into inflamed lymph nodes and tissues^22^. Also, CCR2 and its ligand CCL-2 are molecules extensively studied for their involvement in CNS inflammatory diseases including, multiple sclerosis, Alzheimer’s disease and ischemic stroke^23^. In these previously published studies, CCR2 appears as one of the major molecules required for inflammatory cell homing to the CNS. This suggests that also in some ALS patients CCR2 could be involved in DC recruitment at the diseased tissue.

The decrease of circulating DC numbers and the increased CD62L expression suggests DCs are actively recruited at the inflamed CNS in ALS patients.

### Peripheral blood DCs from a sub-population of ALS patients produce higher amounts of IL-8 and CCL-2 compared to controls

**Figure 2:**
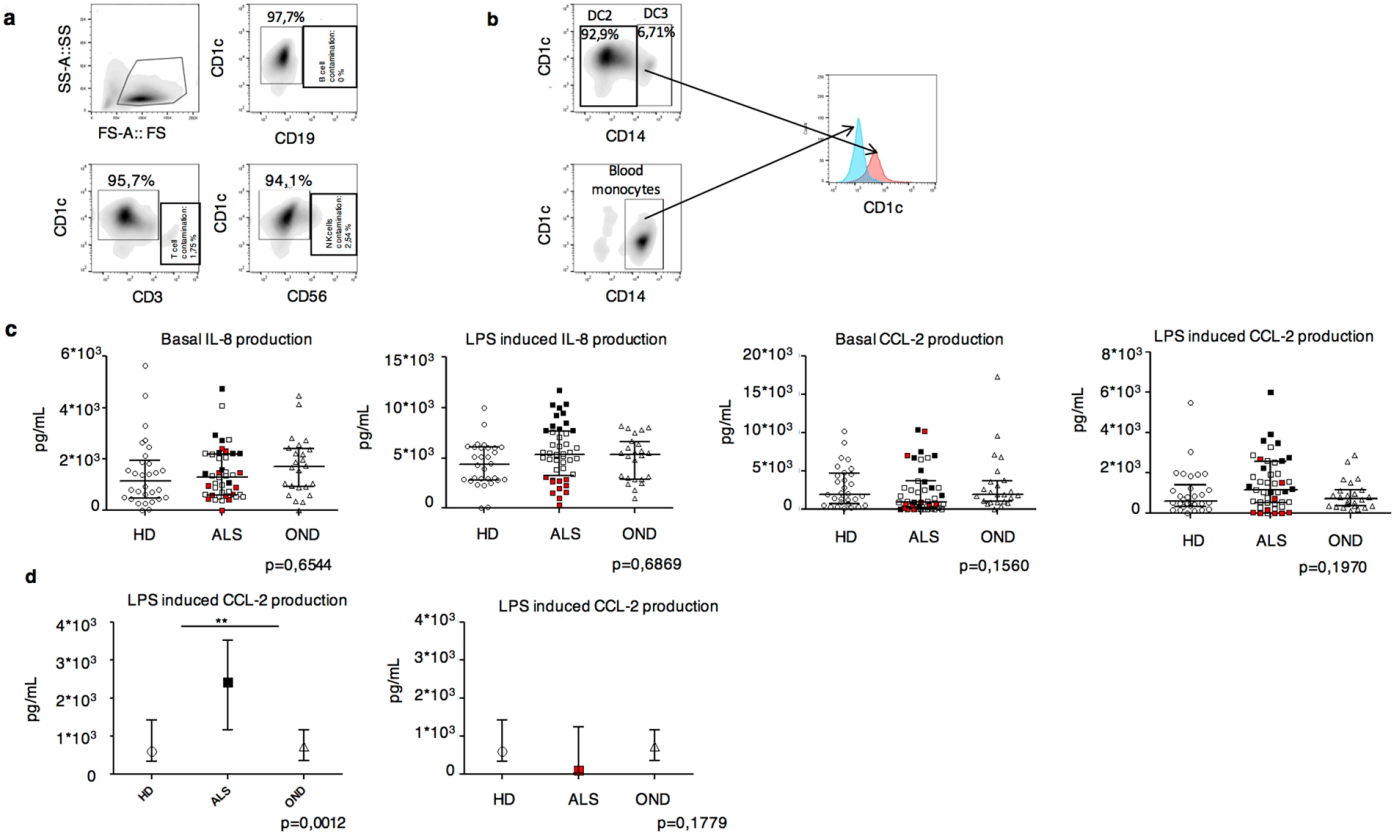
Amounts of spontaneous or LPS-induced chemokine production by DCs purified from peripheral blood of ALS patients (n = 52), HD (n = 36) or OND (n = 25). (**a**) Cytofluorimetric analysis of sorted DCs. The percentages of CD1c^high^ cells and of the T, B and NK cell contaminants are shown. (**b**) CD1c expression levels shown by CD14^+^ DCs and CD14^+^ monocytes. Notice that the two cell types can be distinguished based on the levels of CD1c expression. (**c**) Scatter plots of the amounts of IL-8 and CCL-2 produced by DCs from ALS, HD and ODN 24 h after *in vitro* culture in the presence or absence of LPS. Black dots in LPS-induced IL-8 plots (second to the left panel) are the selected patients with high efficiency of IL-8 production, red dots represent patients with low IL-8 production. (**d**) Efficiency of LPS-induced CCL-2 production by DCs from the selected high IL-8 producers. Each dot represents a patient. **P < 0.01.

Since we observed the upregulation of receptors involved in DC migration, we, next, tested whether the capacity of patients’ DCs to produce chemokines involved in inflammatory cell recruitment was also altered. We therefore analyzed the production of interleukin (IL)-8 (important for neutrophil recruitment), and CCL-2 (a key chemokine for the recruitment of inflammatory cells at the CNS). CD1c^high^ DCs were purified from peripheral blood (Fig. 2a) and cultured in the presence or not of LPS. Purified CD1c^high^ contained two DC sub-populations recently classified as DC2 (CD1c^high^CD14^−^) and DC3 (CD1c^high^CD14^+^)^21^. DC3 were previously erroneously considered monocytes. DC3 differ from monocytes because they express high levels of CD1c, while monocyte express intermediate levels (Fig. 2b)^24^. LPS was chosen as a prototypic inflammatory agent and because of the increased levels of this ligand in the blood of ALS patients. The amount of cytokine released in the supernatant was then measured 24 hours later. Characteristics of ALS patients, HD and OND patients used for this analysis are resumed in Supplementary Tables 3–5.

We first analyzed the total unselected ALS population and we did not observe a significant difference in chemokine production among ALS patients, HD and OND (Fig. 2c,d). Nevertheless, we observed that ALS patients clustered in three distinct sub-populations with a sub-population (Supplementary Table 6), positioned in the fourth quartile, showing an unusual very high LPS-induced production of IL-8 (Fig. 2c). We focused our analysis on these patients and we found they also showed a significant higher efficiency of CCL-2 production compared to controls (Fig. 2d). This behavior was specific for CCL-2, since all the other cytokines tested did not show this segregated distribution (Supplementary Figure 1).

These results suggest that in a subpopulation of ALS patients, DCs can contribute to the inflammatory process by producing inflammatory chemokines, such as CCL-2 and IL-8, in the inflamed lymph nodes and CNS, and contributing to the recruitment of inflammatory cells, i. e. T cells.

We then investigated whether there was a correlation between the efficiency of IL-8 and CCL-2 production by ALS patients’ DCs before and after LPS exposure and several clinical disease parameters, including: i) site of onset, ii) non-invasive mechanic ventilation (NIV), iii) radiological inserted gastrostomy/percutaneous endoscopic gastrostomy (RIG/PEG), iv) ALS-Functional Rating Scale-Revised (alsfrs-r), v) disease progression, vi) alsfrs-r bulbar, vii) alsfrs-r respiratory, viii) alsfrs-s spinal, ix) time from onset to diagnosis, x) time from onset to evaluation and, xi) time from diagnosis to evaluation. The correlations between disease parameters and cytokine production were analyzed with the Spearman test in univariate models (Supplementary Tables 7 and 8). The correlations with a p-value < 0,2 were analyzed again in a multivariate model adjusting for age and sex. We were not able to identify any correlation (data not shown), which is presumably due to the high disease heterogeneity. Nevertheless, high levels of CCL-2 have been shown in the spinal cord of SOD mice^25^, a mouse model of a subclass of ALS disease, and in some ALS patients^26, 27^. Therefore, the altered efficiency of chemokine production by DCs could impact on neuroinflammation in a subpopulation of ALS patients that can be identified by peripheral blood analysis.

### Peripheral blood DCs from ALS patients do not show any difference in the production IL-12p40, TNF-α, IL-1β, IL-6 and IL-10 compared to controls

We then investigated whether DCs in ALS patients contribute to neuroinflammation also *vi*a an altered production of classical inflammatory or anti-inflammatory cytokines. CD1c^+^ DCs were tested for their capacity to produce IL-12p40, TNF-α, IL-1β, IL-6 and IL-10 spontaneously or after LPS challenge.

**Figure 3:**
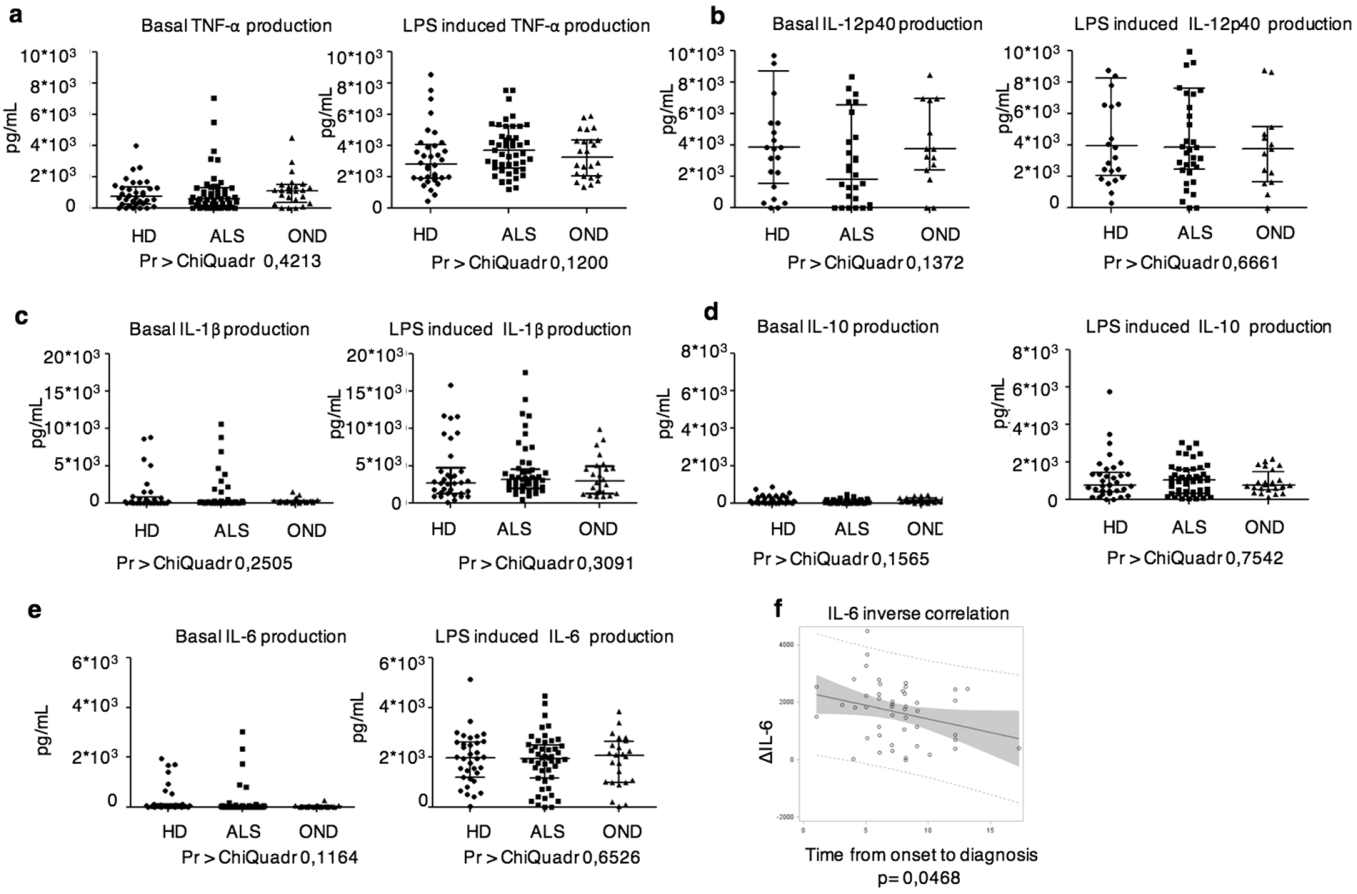
Amounts of spontaneous or LPS-induced cytokine production by DCs purified from peripheral blood of ALS patients (n = 52), HD (n = 36) or OND (n = 25). (**a**–**e**) Scatter plots of the amounts of the indicated cytokines produced by DCs from ALS, HD and ODN 24 h after *in vitro* culture in the presence or absence of LPS. (**f**) IL-6 and Time from Onset to Diagnosis correlation. An inverse correlation (p = 0,048 - Spearman test) between the time from onset to diagnosis and the ΔIL-6 levels (IL-6 LPS – IL-6 NT) is shown.

No significant differences were observed in the spontaneous or LPS induced release of IL-12p40, TNF-α, IL-1β, IL-6 and IL-10 among ALS patients, HD and OND (Fig. 3a–e). Also, analyzing the selected population expressing high amounts of IL-8 and CCL-2 no significant alteration in the expression of these cytokines was found. We then investigated whether there was a correlation between the amounts of single cytokine produced by ALS DCs before and after LPS exposure and the disease parameters as described above. Although many parameter were statistically significant in the univariate analysis (Supplementary Tables 9–12), no significant correlations were found with the multivariate analysis (data not shown) except a significant inverse correlation between the time from onset to diagnosis and the ΔIL6 levels (the amount of IL6 produced after LPS stimulation minus the amount of IL6 spontaneously produced) (Fig. 3f and Supplementary Tables 12 and 13).

**Figure 4:**
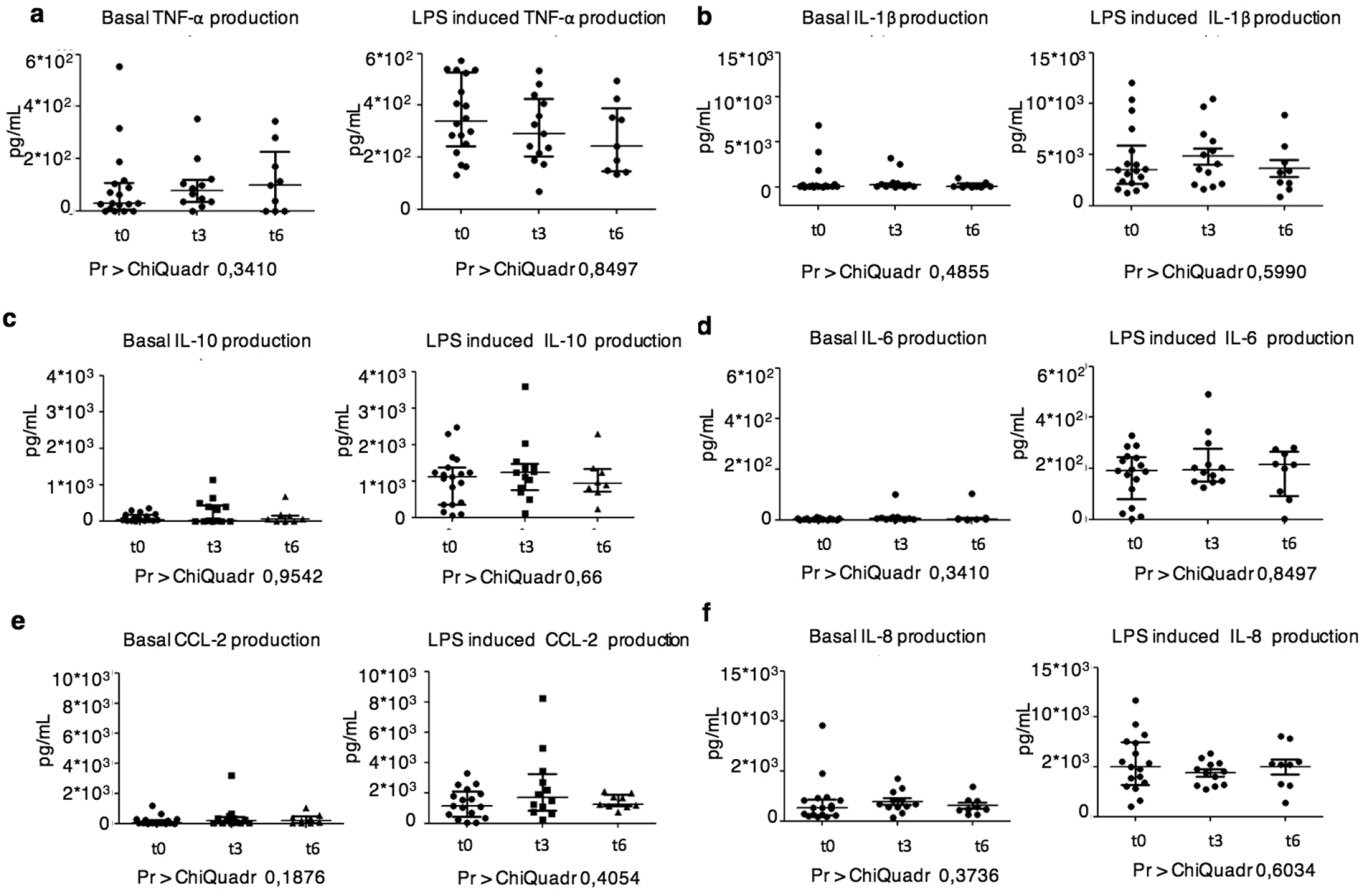
Longitudinal analysis of cytokine production. Scatter plots of the amounts of the indicated cytokines produced by DCs from ALS, HD and ODN 24 h after *in vitro* culture in the presence or absence of LPS. Three time points are shown, the first measure (t0) and the analyses performed 3 months (t3) and 6 months (t6) after the first test.

A longitudinal assay of cytokine production by peripheral blood DCs was performed to investigate possible variations during disease progression. The efficiency of cytokine production remained relatively constant over time (Fig. 4).

Collectively these results suggested DC-derived IL-6 may have a potential inflammatory role in the CNS or in the inflamed lymph node during the initial disease phases. Diversely, DCs do not contribute to the inflammatory process in ALS patients by over-producing the classical inflammatory cytokines or down-modulating the production of anti-inflammatory cytokines.

## Discussion

Several published data highlight the contribution of chronic neuroinflammation in the degenerative process of ALS. Inflammatory T cells infiltrate the CNS of ALS patients and by interacting with glial cells contribute to motor neuron degeneration^28^. To differentiate into inflammatory cells and to acquire the ability to infiltrate diseased tissues, T cells need to interact with professional antigen presenting cells (APCs). Among APCs, myeloid DCs are the most efficient cells in priming T cells and skewing their responses toward an inflammatory or anti-inflammatory phenotype^29^. For this reason, DCs are often deregulated in inflammatory conditions, including neurodegenerative diseases^30^.

The identification of any possible alteration in the functionality of DCs in ALS patients may help defining the origin of inflammation that is important to drive neurodegeneration. Here, we have investigated the phenotype and functionality of circulating DCs in a large cohort of ALS patients. *Ex vi*vo analyses of the frequency, the expression of costimulatory, MHC and migratory molecules of the CD1c^+^ DC subset as well as their ability to produce inflammatory and anti-inflammatory cytokines spontaneously and in response to LPS has been investigated.

We found ALS patients have a significant decrease in the number of circulating DCs. Also, these DCs present an increased expression of CD62L compared with healthy donors. We identified a subpopulation of ALS patients with higher LPS-induced IL-8 and CCL-2 production. Ultimately, we identified a significant inverse correlation between the time from onset to diagnosis and the levels of IL-6 secretion after LPS challenge.

The L-selectin CD62L is a homing leukocytes’ receptor for entering secondary lymphoid tissues, via high endothelial venules, or inflamed tissues^22^. The prediction of an increased recruitment of circulating DCs to the CNS is also supported by the decrease of peripheral blood DC numbers observed in ALS patients compared to healthy donors.

Our data support published results showing mRNAs coding mature and immature DC markers in the spinal cord of ALS patients, and the presence of CD1a^+^CD83^+^ perivascular cells in the degenerating corticospinal tract of ALS patients^9^.

The finding of the upregulation of the levels of CD62L in ALS patients DCs compared to controls is surprising since a downregulation this molecule has been, instead, described in PBMCs and monocytes in Westerners Indians and Japanese patients^31, 32^.

Downregulation of CD62L and CCR2 by classical monocyte described in literature has been interpreted as a dysregulation in the homing capacity of these leukocytes and a patient compensatory response to avoid further inflammation at the diseased tissue. Our observation of CD62L upregulation in DCs argues in favor of a pivotal inflammatory role of these cells during the course of the disease.

A subpopulation of ALS patients also present an overproduction of IL-8 and CCL-2 in response to LPS. CCL2 is a potent chemoattractant expressed mostly by astrocytes, but also by neurons, microglia. CCL-2 attracts CCR2/4-expressing leukocytes, including T cells^33^. According with our results, an increase in CCL-2 expression by DCs has been described in mSOD1 mice. The CCL-2 increased expression is seen before any signs of disease and may indicate the presence of injured cells as early as 15 days of age^34^. In patients, an increase of CCL-2 amounts in the cerebrospinal fluid (CSF) and spinal cord of ALS compared with controls has been reported^9^. Our observations suggest DC recruited from peripheral blood can be an additional source of CCL-2 in the CNS of ALS patients.

IL-8 is a potent chemotactic signaling factor for recruitment of neutrophils to inflamed tissues. Higher amounts of IL-8 have been found in the CSF and serum from ALS patients compared to HD and individuals with non-inflammatory diseases, with considerably higher amount in the CSF compared to serum^11, 35^. A correlation with serum IL-8 levels and disease progression has been also described^36, 37^.

The deregulated IL-8 production by DCs in a subpopulation of ALS patients suggests DCs may represent another source of IL-8 in the inflamed CNS in addition to microglial cells. Given the high amounts of IL-8 released by DCs from some ALS patients we can predict that also neutrophils efficiently contribute to neuroinflammation.

The CD62L overexpression by peripheral inflammatory DCs could be exploited as a target molecule for disease treatment. To date there are no authorized clinical treatment for ALS with anti-CD62L drugs. In the clinic, the blocking anti-CD62L antibody Natalizumab has been tested in the treatment of relapsing and remitting multiple sclerosis^38^ with positive results. A reduction of the annual relapse rate in two clinical trials by 68% has been observed^39, 40^. Considering that Natalizumab could reduce the recruitment at the inflammatory site of all CD62L positive leukocytes, a positive prediction on ALS symptom reduction could be envisaged.

In the present work, we have also found an inverse correlation between the time from onset to diagnosis and the levels of IL6 secretion induced by LPS. The role of IL-6 signaling in neurological diseases has not been completely clarified and may vary depending on the disease stages, on the ratio of free IL-6 and sIL-6R or other factors specific for each disease^41^.

The humanized anti-IL-6R antibody tocilizumab inhibits IL6 signaling through both IL-6R and sIL6-R. Tocilizumab has shown a favorable long-term effect in patients with rheumatoid arthritis^42^ and is under study in patients with Castleman’s disease, juvenile rheumatoid arthritis and inflammatory bowel disease^43^.

An *in vivo* trial of Tocilizumab efficacy in ALS treatment has been recently conducted^44^. Only patients showing a high proinflammatory profile showed a dramatic downmodulation of their inflammatory state with attenuation in the disease progression observed only in a minimal number of patients. The effects were demonstrated to be individual and time- and dose dependent^45^. Our observation of an inverse correlation between the time from onset to diagnosis and the levels of IL6 secretion induced by LPS deserves further investigation on a possible involvement of IL-6 in promoting neurodegeneration only during the very early disease stages. This may explain the difficulties in the interpretation of the anti-IL6R trial results. It is conceivable that an intervention on the IL-6-IL6R axis only during the initial symptomatic phases could be of some efficacy.

In conclusion, an analysis of peripheral blood DCs can be useful to identify ALS patients with an altered course of inflammatory cell recruitment at the diseased tissue. The high levels of CD62L expression by peripheral blood DCs indicate this molecule as a possible new target for *in vivo* treatment to reduce CNS inflammation.

## Material and Methods

### Patients’ enrollment

We studied 72 patients with ALS, consecutively recruited from the ALS Centre at the NEuroMuscular Omnicentre (NEMO). The diagnosis of ALS was based on a detailed history and physical examination, and supported by the electrophysiological evaluation. Other causes of disease were excluded by appropriate blood tests and neuroimaging. ALS patients showed the presence of combined UMN and LMN involvement in at least one body region, and were classified with definite or probable diagnosis according to the revised El Escorial criteria^20^.

All subjects were free of any infection and inflammatory or allergic reaction for at least 2 weeks prior to study admittance. Acute phase reactants (ESR, C-reactive protein, alpha 2-globulins) and rheumatoid factor were negative at blood tests. All patients were taking over 6 months Riluzole, but no therapy with potentially anti-inflammatory properties was administered at the time of the study. The ALS-Functional Rating Scale-Revised (ALS-FRS-R) was used to assess the expression of disease that was rated as follows: severe, with ALS-FRS-R score lower than 28; mild, with ALS-FRS-R score ranged from 29 to 43; and slight, with ALS-FRS-R score upper than 44. Rapidity of disease progression was assessed using the ratio between the score lost on the ALS-FRS-R and the disease duration: Progression index = (48 − ALS-FRS-R score)/duration in months.

Healthy Donors (n = 37) and non-ALS Neurological Patients (n = 25) stratified for age and sex were also enrolled as control.

Supplementary Tables 1–6 resume the characteristics of ALS patients and controls enrolled in the study.

All methods were carried out in accordance with relevant guidelines and regulations. All experimental protocols were approved by the Ethics Committees of NEMO and University of Milano-Bicocca. Informed consent was obtained from all subjects.

### CD1c^+^ DC purification and culture

Blood samples from ALS patients, were obtained after written informed consent. From all subject whole blood was collected by venopuncture in vacutainer tubes containing ethylenendiamine tetra-acetic acid (EDTA; Becton Dickinson, Rutheford, NJ).

DC purification was performed from 18 ml of peripheral blood using CD1c (BDCA-1) Dendritic Cells Isolation kit purchased from Miltenyi Biotech (purity 90%). Cell contaminants were defined by evaluating the presence of B, T, NK cells and monocytes in the purified population. Purified CD1c^high^ DCs (30.000) were plated in RPMI (Euroclone) medium containing g 10% heat-inactivated fetal bovine serum (Gibco, cat. 10270), 100 IU of penicillin, 100 µg/ml of streptomycin, 2mM l-glutamine (all from Euroclone) complete medium and cultured in the presence or absence of 100 ng/mL LPS for 24 hours in 96 well suspension u-bottom plates. The supernatants were than collected for ELISA assays.

### Flow cytometry

After red blood cell lysis, single cell suspensions of blood samples (15 ml) from ALS patients and healthy donors were pelleted, resuspended with the appropriate amount of antibody in 200 μl of PBS, and incubated for 20 min on ice in the dark. Cells were than washed once with 1 ml of PBS and resuspended in a final volume of 270 μl of PBS. Finally, 30 μl of Beckman Coulter Flow-Count Fluorospheres have been added to the samples to determine absolute cells numbers. Peripheral blood DCs have been gated on CD1c^high^CD19^−^ cells. Monocytes were excluded based on side and forward scatter parameters.

For cytofluorimetric analysis, the antibodies used were as follows: anti-CD1c-PE (L161), anti-CD19-APC (HI19), anti-CCR5-alexa700 (HEK/1/85a), anti-HLA DR/DP/DQ-FITC (Tu39), anti-HLA A/B-Pacific Blue (W6/32), anti-CCR7-APC,-Cy7 (G04347), anti-CCR2-PECy7 (KO36C2), anti-CD80-FITC (2D10), anti-CD86-Pacific Blue (IT2.2), anti-CD62L Alexa700 (DREG-56), anti-CD40-PECy7 (5C3); anti-CD3-PEcy7 (HIT3a), anti-CD1c-APCcy7 (L161), anti-CD14-PE (HCD14), anti-CD19-FITC (HIB19), anti-CD56-APC (NCAM) all from Biolegend. Samples were acquired with a Gallios flow cytometer (Beckman-Culter) and analyzed with FlowJo (TreeStar) software.

### ELISA assay

Concentrations of TNF-α, IL-1β, IL-6, IL-12p40, IL-8, CCL2 and IL-10 in BDCA1^+^ DC supernatants were assessed by ELISA kits purchased from R&D Systems.

### Antibodies and chemicals

Antibodies for flow cytometry were purchased from Biolegend and TRL4-grade smooth LPS (E. coli,O55:B5) was purchased from Enzo Life Sciences.

### Statistical Analysis

Medians were compared by Wilcoxon Test or Kruskal-Wallis Tests. Data are expressed and plotted as median [Q1-Q3]. Sample sizes for each experimental condition are provided in the figures and the respective legends. The correlations between disease parameters and cytokine production were analyzed with the Spearman test in univariate models. The correlations with a p-value < 0,2 were analyzed again in a multivariate model adjusting for age and sex.

### Data availability

Data and associated protocols promptly available to readers.

## Electronic supplementary material

Supplementary information
