# Supplementary material for: Inflammatory role of dendritic cells in Amyotrophic Lateral Sclerosis revealed by an analysis of patients’ peripheral blood
Source: Sci Rep. 2017 Aug 10;7:7853. doi: 10.1038/s41598-017-08233-1 (PMC5552769; doi:10.1038/s41598-017-08233-1)
Supplement: Supplementary file 1 — Supplementary information [file 41598_2017_8233_MOESM1_ESM.pdf]

SUPPLEMENTARY MATERIALS

Inflammatory role of dendritic cells in Amyotrophic Lateral Sclerosis revealed by an analysis of patients' peripheral blood

Michela Rusconi[1], Francesca Gerardi[2], William Santus[1], Andrea Lizio[2], Valeria Ada Sansone[2,4], Christian Lunetta[2], Ivan Zanoni[1,3*&], Francesca Granucci[1*&]

[1] *Department of Biotechnology and Bioscience, University of Milano-Bicocca, Piazza della Scienza 2, 20126 Milan, Italy*
[2] *NEuroMuscolar Omnicentre, Fondazione Serena Onlus, Milano, Italy*

[3] *Harvard Medical School and Division of Gastroenterology, Boston Children's Hospital, Boston, MA 02115, USA*
[4] *Dept. Biomedical Sciences for Health, University of Milan, Milan, Italy.*

& equal contribution

* corresponding authors:

Francesca Granucci,
Department of Biotechnology and Biosciences, University of Milano-Bicocca, Piazza della Scienza 2 20126 Milan, Italy.
Phone: +390264483553
Fax: +390264483552
e-mail: francesca.granucci@unimib.it

Ivan Zanoni,
Division of Gastroenterology, Boston Children's Hospital, Enders Building 675, Longwood Ave 300, Boston, MA 02115, USA.
Phone: +16179196123
e-mail: ivan.zanoni@childrens.harvard.edu

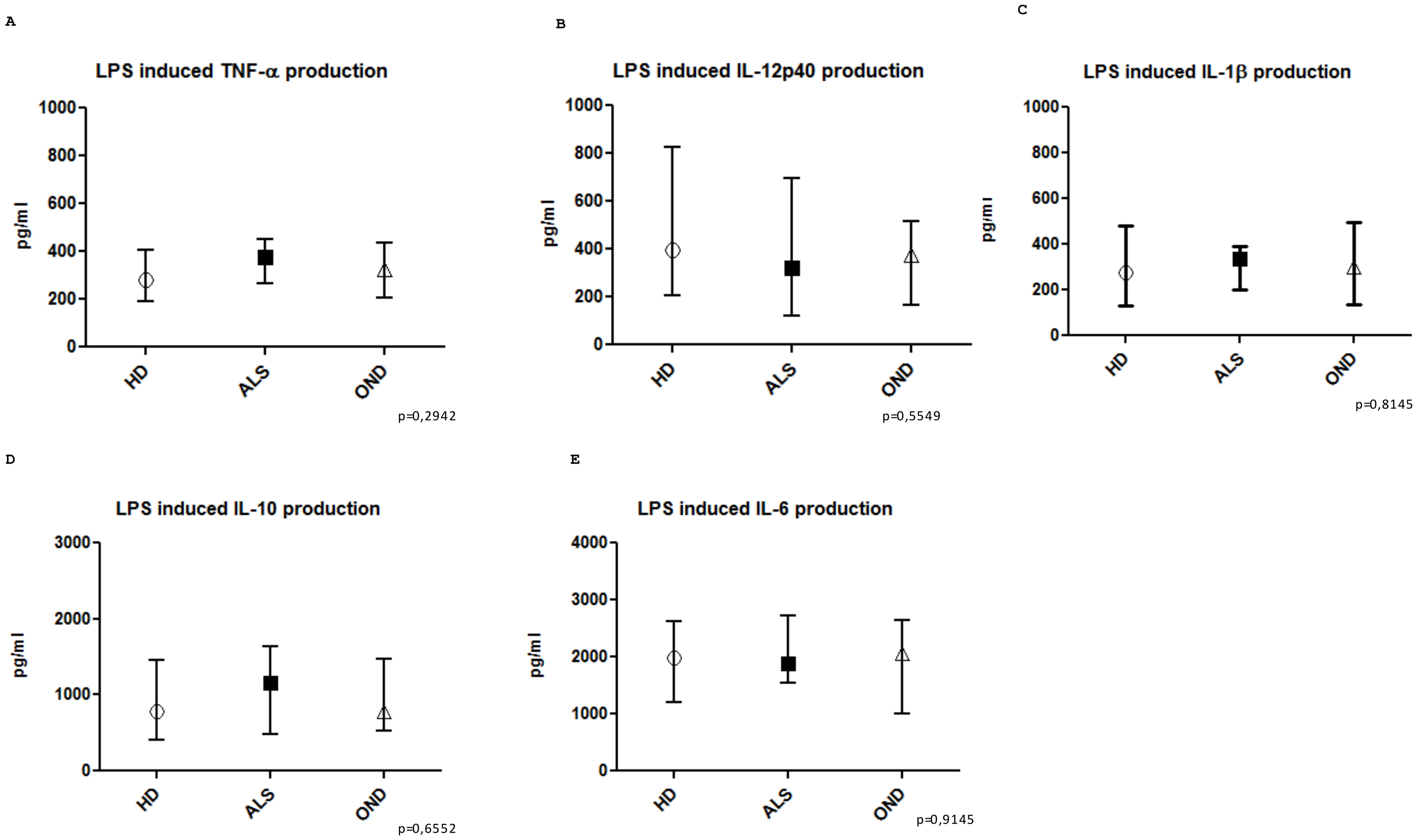

Supplementary Figure 1. (A-E) Efficiency of the indicated cytokine production by DCS from high IL-8 producer ALS patients after LPS treatment.

Supplementary Table 1 ALS patients for flow cytometry analysis

	Sex	Date Of Birthday	Onset	Type of Onset	Diagnosis	Progression Index	El Escorial	ALSFRS/48	ALSFRS Bulbar	ALSFRS Respiratory
ALS100	M	16/09/1949	01/10/2013	Spinal	30/01/2014	1,29	Probable	36	9	11
ALS101	M	28/04/1947	01/01/2014	Spinal	01/06/2014	0,84	Probable	20	8	6
ALS102	M	03/03/1952	01/01/2005	Spinal	01/10/2006	0,15	Probable	18	4	6
ALS103	M	14/01/1944	01/09/2010	Spinal	01/01/2012	0,23	Probable	12	9	3
ALS104	M	23/06/1971	01/09/2014	Spinal	01/12/2014	1,60	Defined	29	9	11
ALS105	F	10/11/1962	01/10/2008	Spinal	01/09/2009	0,55	Probable	45	11	12
ALS106	F	22/02/1950	01/12/2014	Spinal	01/05/2015	3,52	Probable	46	12	12
ALS107	M	30/01/1947	01/11/2013	Bulbar	01/01/2014	0,89	Probable	26	9	3
ALS108	M	18/08/1953	01/10/2014	Spinal	01/02/2015	1,55	Defined	25	9	12
ALS109	M	24/09/1963	01/01/1993	Spinal	01/01/2009	0,04	Defined	4	0	4
ALS110	F	09/09/1947	01/12/2013	Spinal	01/07/2014	1,11	Probable	26	11	6
ALS111	F	08/04/1941	01/06/2013	Bulbar	01/12/2013	0,30	Defined	9	1	6
ALS112	M	25/10/1952	01/10/2014	Bulbar	01/10/2015	5,10	Probable	43	10	12
ALS113	M	24/04/1949	01/01/2012	Spinal	01/05/2014	1,13	Probable	29	11	12
ALS114	F	15/06/1958	01/01/2006	Spinal	01/01/2007	0,07	Defined	8	5	3
ALS115	F	29/04/1945	01/01/2009	Spinal	01/06/2011	0,26	Probable	16	6	10
ALS116	M	18/10/1952	01/01/2012	Spinal	01/10/2013	0,36	Defined	12	4	6
ALS117	M	20/10/1965	01/09/2015	Spinal	01/12/2015	6,43	Probable	42	10	12
ALS118	M	20/07/1943	01/06/2008	Spinal	01/10/2010	0,24	Probable	17	9	3
ALS119	M	24/06/1947	01/08/2015	Spinal	01/06/2016	35,56	Probable	32	12	12

Supplementary Table 2 HD for flow cytometry analysis

	sex	Date of Birthday
HD100	F	08/09/1960
HD101	F	15/05/1949
HD102	M	01/05/1950
HD103	F	27/07/1955
HD104	F	26/03/1950
HD105	F	02/03/1960
HD106	M	10/02/1959
HD107	F	15/05/1976
HD108	M	22/03/1983
HD109	F	03/02/1958

Supplementary Table 3. ALS patients for cytokine production analysis

	Sex	Date Of Birthday	Onset	Type of Onset	Diagnosis	Progression index	El Escorial	ALSFRS/48	ALSFRS Bulbar	ALSFRS Respiratory
ALS1	F	28/10/1931	01/09/2014	Bulbar	01/03/2015	17,73	Probable	26	4	7
ALS2	M	23/12/1938	01/09/2014	Spinal	01/03/2015	21,14	Defined	31	6	12
ALS3	M	18/02/1953	01/09/2014	Spinal	01/03/2015	18,10	Defined	35	12	12
ALS4	F	17/12/1956	01/04/2013	Bulbar	01/04/2014	2,29	Defined	30	6	7
ALS5	M	18/10/1934	01/01/2014	Bulbar	01/10/2014	2,29	Probable	16	5	3
ALS7	F	26/02/1944	01/06/2013	Bulbar	01/02/2014	0,58	Defined	9	1	5
ALS8	F	19/02/1953	01/06/2014	Bulbar	01/02/2015	12,06	Probable	43	9	12
ALS9	M	09/03/1939	01/03/2014	Spinal	01/11/2014	5,24	Probable	36	11	11
ALS10	F	23/03/1948	01/05/2013	Spinal	01/01/2014	0,71	Probable	12	5	7
ALS11	F	08/04/1941	01/06/2013	Bulbar	01/01/2014	1,29	Probable	22	3	6
ALS12	F	16/03/1938	01/12/2013	Bulbar	01/06/2014	1,55	Probable	20	5	6
ALS13	F	03/07/1954	01/12/2013	Bulbar	01/10/2014	2,71	Defined	24	5	5
ALS14	M	25/09/1953	01/01/2015	Bulbar	01/05/2015	21,00	Probable	42	10	11
ALS15	M	05/09/1972	01/09/2013	Bulbar	01/06/2014	3,02	Defined	40	10	12
ALS16	F	15/01/1957	01/07/2014	Bulbar	01/12/2014	3,15	Defined	23	4	10
ALS17	M	06/03/1952	01/09/2013	Bulbar	01/09/2014	3,10	Defined	32	8	6
ALS22	M	13/11/1941	01/07/2013	Respiratory	01/10/2014	3,07	Probable	31	6	7
ALS24	F	04/05/1939	01/01/2014	Bulbar	01/01/2015	2,65	Probable	23	5	6
ALS27	F	01/07/1966	01/05/2014	Spinal	01/11/2014	3,28	Defined	37	9	12
ALS28	F	19/02/1962	01/03/2014	Spinal	26/10/2014	3,20	Defined	37	12	12
ALS29	M	08/07/1954	01/08/2014	Spinal	01/04/2015	4,42	Defined	28	9	12
ALS30	M	16/12/1940	01/06/2014	Bulbar	01/02/2015	4,61	Defined	39	9	9
ALS31	F	10/01/1942	01/07/2014	Bulbar	01/04/2015	4,31	Defined	28	6	6
ALS32	M	20/08/1967	01/12/2013	Spinal	01/01/2014	1,48	Probable	32	12	12
ALS33	F	30/04/1949	01/02/2015	Spinal	01/07/2015	10,37	Probable	37	12	11
ALS34	F	06/01/1947	01/06/2014	Spinal	01/07/2015	9,00	Defined	33	9	12
ALS35	M	07/11/1950	01/10/2013	Spinal	01/01/2014	1,63	Probable	36	10	11
ALS37	M	23/02/1940	01/05/2014	Bulbar	01/05/2015	3,25	Defined	22	6	3
ALS40	F	03/07/1973	01/02/2014	Spinal	01/07/2014	2,38	Defined	41	12	12
ALS41	M	20/08/1967	01/05/2015	Spinal	01/07/2015	6,32	Defined	32	11	12
ALS42	M	26/03/1943	01/12/2014	Spinal	01/06/2015	1,95	Probable	12	9	1
ALS43	M	06/03/1970	01/03/2013	Spinal	01/10/2013	1,51	Probable	40	12	11
ALS46	M	23/06/1971	01/12/2013	Spinal	01/12/2014	2,28	Defined	29	11	10
ALS47	M	16/10/1952	01/03/2015	Spinal	01/04/2015	1,37	Probable	14	11	1
ALS48	M	28/04/1966	01/04/2015	Spinal	01/11/2015	12,81	Defined	41	11	12
ALS49	M	31/10/1950	01/11/2014	Spinal	01/07/2015	1,59	Defined	12	8	3
ALS50	F	18/02/1944	01/03/2015	Bulbar	01/08/2015	5,69	Defined	37	8	10
ALS51	M	09/08/1956	01/12/2014	Spinal	01/07/2015	4,43	Probable	34	11	12
ALS52	M	24/06/1954	01/12/2014	Spinal	01/04/2015	3,84	Probable	42	12	12

Supplementary Table 4. HD for cytokine production analysis

	Sex	Date Of Birthday
HD1	F	2/26/1944
HD2	M	8/8/1946
HD3	F	7/2/1951
HD 4	F	11/9/1940
HD5	F	9/19/1956
HD6	M	11/1/1939
HD7	F	12/3/1956
HD8	F	6/17/1949
HD9	M	10/10/1949
HD10	F	5/11/1971
HD11	F	11/22/1957
HD12	M	1/25/1967
HD13	F	10/23/1956
HD14	F	8/20/1963
HD15	M	7/26/1965
HD16	F	5/8/1956
HD17	F	12/2/1964
HD18	M	3/30/1968
HD19	F	8/21/1953
HD20	F	7/12/1956
HD21	M	2/23/1958
HD22	F	11/11/1971
HD23	F	7/10/1956
HD24	F	2/6/1960
HD25	F	4/24/1943
HD26	F	6/14/1979
HD27	F	12/28/1981
HD28	M	9/21/1942
HD29	F	3/7/1950
HD30	M	4/15/1950
HD31	M	12/13/1961
HD32	M	5/11/1973
HD33	F	4/18/1953
HD34	F	12/17/1955
HD35	F	4/18/1981
HD36	M	12/16/1947
HD37	F	7/27/1955

Supplementary Table 5. OND for cytokine production analysis

	Sex	Date of Birthday	Disease
OND1	F	7/16/1971	TARLOV CYST
OND2	F	9/5/1959	TARLOV CYST
OND3	F	8/29/1962	TARLOV CYST
OND4	M	7/29/1966	STRUMPEL – LORRAIN DISEASE
OND5	F	5/18/1937	PERIPHERAL POLYNEUROPHATY
OND6	M	11/4/1969	DUCHENNE MUSCULAR DISTROPHY
OND7	F	6/3/1948	MYOTOCHONDRIAL MYOPATHY
OND8	F	8/17/1973	MYOTONIC DISTROPHY TYPE I
OND9	F	1/13/1969	MYOTONIC DISTROPHY TYPE I
OND10	M	7/23/1978	MYOTONIC DISTROPHY TYPE I
OND11	F	1/3/1979	FASCIOSCAPIULOHUMERAL MUSCULAR DYSTROPHY
OND12	M	2/25/1967	MYOTONIC DISTROPHY TYPE II
OND13	M	7/29/1973	MYOTONIC DISTROPHY TYPE I
OND14	F	7/12/1962	MYOTONIC DISTROPHY TYPE II
OND15	M	1/15/1966	MYOTONIC DISTROPHY TYPE I
OND16	F	3/15/1956	TARLOV CYST
OND17	M	4/24/1970	MYOTONIC DISTROPHY TYPE I
OND18	M	7/6/1982	MYOTONIC DISTROPHY TYPE II
OND19	M	3/3/1978	MYOTONIC DISTROPHY TYPE I
OND20	F	11/11/1975	FASCIOSCAPIULOHUMERAL MUSCULAR DYSTROPHY
OND21	F	6/2/1955	MYOTONIC DISTROPHY TYPE I
OND22	M	1/14/1971	MYOTONIC DISTROPHY TYPE I
OND23	M	11/5/1949	TARLOV CYST
OND24	M	6/3/1949	MYOTONIC DISTROPHY TYPE I
OND25	F	10/26/1963	MYOTONIC DISTROPHY

Supplementary Table 6. ALS patients high IL-8 producers

	sex	Date Of Birthday	Onset	Type of onset	Diagnosis	Progression index	El Escorial	ALSFRS/48	ALSFRS Bulbar	ALSFRS Respiratory
ALS18	F	30/08/1944	01/07/2013	Spinal	01/12/2014	5,39	Probable	43	12	12
ALS19	M	06/07/1953	01/10/2014	Spinal	01/07/2015	41,11	Probable	37	12	12
ALS20	M	28/05/1955	01/11/2013	Spinal	01/04/2014	2,47	Probable	40	12	12
ALS21	M	04/05/1978	01/05/2014	Spinal	01/01/2015	4,27	Probable	30	9	11
ALS23	M	24/01/1951	01/12/2014	Spinal	01/04/2015	3,18	Probable	18	7	6
ALS25	M	02/10/1962	01/04/2014	Bulbar	01/11/2014	1,01	Defined	11	4	6
ALS26	M	18/08/1953	01/10/2014	Spinal	01/02/2015	4,21	Defined	33	11	12
ALS36	M	27/02/1944	01/06/2014	Spinal	01/11/2014	1,02	Probable	13	7	6
ALS38	M	14/03/1953	01/11/2014	Spinal	01/05/2015	5,91	Defined	40	12	12
ALS45	M	02/09/1948	01/12/2013	Spinal	01/07/2014	2,37	Probable	41	11	12

Supplementary Table 7

IL-8 Univariate Analysis		NT	LPS	LPS-NT
**Gender**	*rho*	0,06065	0,27752	0,25546
	p-value	0,6957	0,0682	0,0942
**Site Of Onset**	*rho*	-0,20769	-0,32898	-0,28119
	p-value	0,1761	**0,0292**	0,0645
**NIV**	*rho*	0,04651	-0,18603	-0,253
	p-value	0,7643	0,2266	0,0975
**RIG/PEG**	*rho*	-0,07846	-0,20987	-0,26479
	p-value	0,6127	0,1715	0,0824
**Age**	*rho*	0,02282	-0,11315	-0,13304
	p-value	0,8831	0,4646	0,3893
**Alsfrs-r**	*rho*	-0,04378	0,14907	0,21516
	p-value	0,7778	0,3342	0,1607
**Disease Progression**	*rho*	0,02192	-0,12292	-0,16486
	p-value	0,8877	0,4267	0,2849
**Alsfrs-r Bulbar**	*rho*	0,07862	0,2945	0,3019
	p-value	0,6119	0,0523	**0,0464**
**Alsfrs-r Respiratory**	*rho*	0,05932	0,27122	0,30247
	p-value	0,7021	0,0749	**0,0460**
**Time from Onset to Diagnosis**	*rho*	-0,0486	-0,11098	-0,09728
	p-value	0,7540	0,4733	0,5299
**Time from Onset to Evaluation**	*rho*	0,01692	-0,02389	-0,05519
	p-value	0,9132	0,8777	0,7220
**Time from diagnosis to Evaluation**	*rho*	0,01205	-0,03545	-0,05329
	p-value	0,9381	0,8193	0,7312

Supplementary Table 8

CCL-2 Univariate Analysis		NT	LPS	LPS-NT
**Gender**	*rho*	-0,12729	0,05295	0,12705
	p-value	0,4047	0,7298	0,4056
**Site Of Onset**	*rho*	0,12649	-0,06476	-0,11022
	p-value	0,4077	0,6726	0,4710
**NIV**	*rho*	-0,12729	-0,17296	-0,20823
	p-value	0,4047	0,2559	0,1699
**RIG/PEG**	*rho*	0,15553	-0,05914	-0,14784
	p-value	0,3076	0,6995	0,3324
**Age**	*rho*	0,01225	-0,1557	-0,21753
	p-value	0,9363	0,3071	0,1512
**Alsfrs-r**	*rho*	0,0729	0,09605	0,10085
	p-value	0,6341	0,5303	0,5098
**Disease Progression**	*rho*	-0,20007	-0,12889	-0,05001
	p-value	0,1876	0,3988	0,7443
**Alsfrs-r Bulbar**	*rho*	0,08795	0,06912	0,09277
	p-value	0,5656	0,6519	0,5444
**Alsfrs-r Respiratory**	*rho*	0,03252	0,17081	0,18693
	p-value	0,8321	0,2619	0,2189
**Time from Onset to Diagnosis**	*rho*	0,21578	-0,17559	-0,21036
	p-value	0,1546	0,2486	0,1654
**Time from Onset to Evaluation**	*rho*	0,22125	-0,03044	-0,16958
	p-value	0,1441	0,8426	0,2654
**Time from diagnosis to Evaluation**	*rho*	0,088	0,08442	-0,03018
	p-value	0,5654	0,5814	0,8440

Supplementary Table 9

TNF-α Univariate Analysis		NT	LPS	LPS-NT
**Gender**	*rho*	-0,0187	0,00932	-0,02796
	p-value	0,8996	0,9499	0,8504
**Site Of Onset**	*rho*	0,17468	-0,0865	-0,1577
	p-value	0,2350	0,5590	0,2844
**NIV**	*rho*	0,29774	0,20606	-0,02306
	p-value	**0,0398**	0,1600	0,8763
**RIG/PEG**	*rho*	0,31367	0,31108	0,05742
	p-value	**0,0299**	**0,0314**	0,6983
**Age**	*rho*	0,15215	0,12124	-0,05189
	p-value	0,3019	0,4117	0,7261
**Alsfrs-r**	*rho*	-0,3494	-0,1645	0,07367
	p-value	**0,0149**	0,2638	0,6188
**Disease Progression**	*rho*	0,25059	0,17034	-0,01368
	p-value	0,0858	0,2471	0,9265
**Alsfrs-r Bulbar**	*rho*	-0,2284	-0,0348	0,10715
	p-value	0,1184	0,8145	0,4685
**Alsfrs-r Respiratory**	*rho*	-0,3934	-0,0385	0,20514
	p-value	**0,0057**	0,7951	0,1619
**Time from Onset to Diagnosis**	*rho*	0,06583	0,12396	0,06126
	p-value	0,6566	0,4012	0,6791
**Time from Onset to Evaluation**	*rho*	0,13599	-0,021	-0,05819
	p-value	0,3567	0,8876	0,6944
**Time from diagnosis to Evaluation**	*rho*	0,11027	-0,0309	-0,03485
	p-value	0,4556	0,8347	0,8141

Supplementary Table 10

IL-1β Univariate Analysis		NT	LPS	LPS-NT
**Gender**	*rho*	0,01553	-0,02174	0,14289
	p-value	0,9165	0,8834	0,3326
**Site Of Onset**	*rho*	0,05141	0,31426	0,16931
	p-value	0,7286	**0,0296**	0,2500
**NIV**	*rho*	0,14302	0,2414	0,02614
	p-value	0,3322	0,0983	0,8600
**RIG/PEG**	*rho*	0,12444	0,27754	0,14356
	p-value	0,3994	0,0561	0,3303
**Age**	*rho*	0,08706	0,11041	-0,03733
	p-value	0,5563	0,4550	0,8011
**Alsfrs-r**	*rho*	0,02243	-0,04882	0,10667
	p-value	0,8797	0,7418	0,4706
**Disease Progression**	*rho*	0,03917	0,16421	0,06943
	p-value	0,7915	0,2647	0,6391
**Alsfrs-r Bulbar**	*rho*	-0,00052	-0,23166	-0,05995
	p-value	0,9972	0,1131	0,6856
**Alsfrs-r Respiratory**	*rho*	-0,05813	-0,14179	0,06476
	p-value	0,6947	0,3364	0,6619
**Time from Onset to Diagnosis**	*rho*	-0,10739	-0,04869	-0,08536
	p-value	0,4675	0,7424	0,5640
**Time from Onset to Evaluation**	*rho*	-0,05679	-0,20231	-0,277
	p-value	0,7014	0,1679	0,0567
**Time from diagnosis to Evaluation**	*rho*	0,02604	-0,16639	-0,26046
	p-value	0,8606	0,2584	0,0738

Supplementary Table 11

IL12p40 Univariate Analysis		NT	LPS	LPS-NT
**Gender**	*rho*	0,07513	0,17553	0,06036
	p-value	0,6632	0,3058	0,7266
**Site Of Onset**	*rho*	0,06773	0,08127	0,04065
	p-value	0,6947	0,6375	0,8139
**NIV**	*rho*	0,19855	0,49919	0,28372
	p-value	0,2457	**0,0019**	0,0936
**RIG/PEG**	*rho*	0,28771	0,50151	0,215
	p-value	0,0888	**0,0018**	0,2079
**Age**	*rho*	-0,00458	0,16255	0,06194
	p-value	0,9789	0,3435	0,7197
**Alsfrs-r**	*rho*	-0,07048	-0,36607	-0,31536
	p-value	0,6829	**0,0281**	0,0610
**Disease Progression**	*rho*	-0,01208	0,34494	0,35967
	p-value	0,9442	**0,0394**	**0,0312**
**Alsfrs-r Bulbar**	*rho*	-0,05943	-0,21595	-0,14966
	p-value	0,7306	0,2059	0,3837
**Alsfrs-r Respiratory**	*rho*	-0,12673	-0,28625	-0,10109
	p-value	0,4614	0,0905	0,5575
**Time from Onset to Diagnosis**	*rho*	0,05799	-0,07899	-0,00032
	p-value	0,7369	0,6470	0,9985
**Time from Onset to Evaluation**	*rho*	0,22469	-0,02793	-0,18272
	p-value	0,1877	0,8715	0,2861
**Time from diagnosis to Evaluation**	*rho*	0,11625	-0,01693	-0,1324
	p-value	0,4996	0,9219	0,4414

Supplementary Table 12

IL-6 Univariate Analysis		NT	LPS	LPS-NT
**Gender**	*rho*	0,03886	0,00645	0,02581
	p-value	0,7954	0,9657	0,8633
**Site Of Onset**	*rho*	-0,13953	-0,04155	-0,11826
	p-value	0,3496	0,7815	0,4285
**NIV**	*rho*	0,08905	0,00968	-0,0968
	p-value	0,5517	0,9485	0,5174
**RIG/PEG**	*rho*	0,0287	0,16824	0,02355
	p-value	0,8481	0,2583	0,8751
**Age**	*rho*	0,1805	-0,08358	-0,11217
	p-value	0,2247	0,5765	0,4528
**Alsfrs-r**	*rho*	-0,00029	-0,04488	0,0351
	p-value	0,9985	0,7645	0,8148
**Disease Progression**	*rho*	0,00107	0,08344	0,01139
	p-value	0,9943	0,5771	0,9394
**Alsfrs-r Bulbar**	*rho*	0,12222	-0,04393	-0,00356
	p-value	0,4131	0,7694	0,9811
**Alsfrs-r Respiratory**	*rho*	-0,02378	0,16525	0,22808
	p-value	0,8739	0,2670	0,1231
**Time from Onset to Diagnosis**	*rho*	0,02894	-0,21992	-0,27516
	p-value	0,8469	0,1375	0,0612
**Time from Onset to Evaluation**	*rho*	0,05208	-0,07575	-0,10437
	p-value	0,7281	0,6128	0,4851
**Time from diagnosis to Evaluation**	*rho*	-0,01503	0,00202	0,01313
	p-value	0,9201	0,9892	0,9302

Supplementary Table 13

IL-6 Multivariate Analysis	Time from Onset to Diagnosis
**LPS-NT**	p=0,0468
